# Cross-Cultural Chronotypes: Educating Medical Students in America, Malaysia and the UAE

**DOI:** 10.15694/mep.2020.000005.1

**Published:** 2020-01-07

**Authors:** Nicoline Schiess, Violet Kulo, Jennifer L. Dearborn, Sami Shaban, Charlene E. Gamaldo, Rachel Marie E. Salas

**Affiliations:** 1 Johns Hopkins University; 2 Johns Hopkins School of Medicine; 3 Beth Israel Deaconess Department of Neurology; 4 United Arab Emirates University College of Medicine and Health Sciences; 5 Johns Hopkins Department of Neurology

**Keywords:** medical students, sleep, chronotype, sleep education

## Abstract

This article was migrated. The article was marked as recommended.

**Background:** Ample data demonstrates that sleep deprivation leads to impaired functioning including cognitive performance, memory and fine motor skills. Medical students represent a professional sector in which optimizing cognitive performance and functioning is critical from a personal, public health and safety perspective.

**Aims:** To characterize chronotypes of an international cohort of medical students and determine if chronotype is affected by demographics or latitude.

**Samples:** 328 students from medical schools in the United States (US), Malaysia and United Arab Emirates (UAE) were assessed for differences in chronotype and sleep habits.

**Methods:** A cross-sectional, questionnaire-based study from medical schools in the US, Malaysia and UAE between 2013 and 2015.

**Results:** There was a significant difference in mean waking times for Malaysian students who reported awakening earlier than US or UAE students. Malaysian students were most likely to feel their best earlier in the day and consider themselves a “morning type.” UAE students were more likely to do “hard physical work” later in the day, followed by US and Malaysian. On average, US students were less likely to shift their bedtime later if they had no commitments the next day. Overall, mean chronotype score was “neither” type for all three groups however the Malaysian group showed a significant preference for morning hours in some individual questions.

**Conclusion:** Medical student sleep patterns vary internationally but chronotype may not. Improving sleep education globally, with awareness of the effects of chronotype, could ultimately result in improved sleep awareness, potentially influencing physician wellbeing, patient care and safety.

## Introduction

Circadian rhythms, which drive human sleep wake cycles, are primarily driven by the central nervous system. There is ample data demonstrating that sleep deprivation leads to impaired performance in many aspects of functioning including cognitive performance, memory and fine motor skills ( Krause *et al*., 2017, Smith, 1996, Ayalon and Friedman, 2008).

Arguably, physicians represent a professional sector in which steps to optimize cognitive performance and functioning is critical from a personal, as well as public health and safety perspective.

While the potential relationship between intern and resident sleep patterns and the delivery of healthcare has been a considerable topic in the last two decades, the sleep behavior of medical students has been less scrutinized. Multiple surveys have shown that medical students do not obtain the amount of sleep that they feel they need ( Grady and Roberts 2017, Ayala *et al*., 2017, Ahmed, Sadat and Cukor, 2017). A study at SUNY Downstate Medical School showed that only 25% of medical students slept an average of 7 hours per night despite 70% of them feeling they required this amount ( Ahmed, Sadat and Cukor, 2017). Although more knowledgeable about sleep in general, students in their final 2 years of medical school had more sleepiness than those in the preclinical years. A larger study looking at 49 medical schools found similar results of slightly less than 7 hours of sleep per night with students in the second year of school (preclinical) receiving the most amount of sleep ( Ayala *et al.,* 2017).

Personal health behaviors practiced by physicians influence the emphasis they place on healthy lifestyle behaviors in the management and counseling of their own patients ( Bleich *et al*., 2014). A physician’s minimization of sleep behavior can suggest an under emphasis of the importance of their patient’s sleep habits. In other words, a lack of knowledge of healthy sleep behavior and habits, i.e. “sleep hygiene”, can lead to physicians who not only fail to recognize detrimental and potentially dangerous sleep habits within themselves but are also ill equipped to recognize and treat within their patient population.

A large amount of evidence has also demonstrated that sleep is important, indeed essential, for learning ( Curcio, Ferrara and De Gennaro, 2006, Peigneux *et al*., 2001, Diekelmann and Born, 2010, Ferrara *et al*., 2006). The pattern of poor academic performance in students and abnormal sleep habits has been demonstrated in multiple studies both in school aged children as well as college students ( Curcio, Ferrara and De Gennaro, 2006, Preckel *et al.,* 2013, Beebe, Rose and Amin, 2010, Wolfson and Carskadon, 2003) often with early sleep-wake schedules correlating with better academic achievement ( Eliasson, Lettieri and Eliasson, 2010, Wolfson and Carskadon, 2003, Genzel *et al.,* 2013).

Geographical location - more specifically latitude - has been proposed as an explanation for differences in sleep chronotype with the idea that one’s chronotype is the synchronization between an internal biorhythm and external cues such as the light-dark cycle ( Miguel *et al.,* 2014, Pereira, Tufik and Louzada, 2005). The light-dark cycle is influenced by a variety of factors including latitude, sunrise, sunset and levels of solar irradiation ( Leocadio-Miguel *et al.* 2017). One study comparing chronotypes in two cities in Brazil found that students living further away from the equator were more prone to an evening chronotype ( Miguel *et al.,* 2014). A large study using the Horne and Ostberg scale in university students in six countries (USA, England, The Netherlands, Colombia, Spain and India) showed that respondents from countries closer to the equator (Colombia, Spain, India) perceived themselves to be more morning-oriented than those students further away from the equator such as USA, England and The Netherlands ( Smith *et al*., 2002). A large study in Brazil on 12,884 subjects living within the same time zone but different latitudes found that farther distance from the equator line equates with a more significant change towards an evening chronotype ( Leocadio-Miguel *et al.,* 2017).

Although sleep health has increasingly been recognized as a pillar to overall health and wellbeing, inclusion of sleep medicine education in the medical school curriculum is woefully lacking. In 1990, evidence from one survey demonstrated that 37% of medical schools provided no sleep education ( Owens, 2005). Internationally, the amount of sleep education is even less. A survey of 409 medical schools in 12 countries (Australia, India, Indonesia, Japan, Malaysia, New Zealand, Singapore, South Korea, Thailand, United States, Canada and Vietnam) showed a similar average of just under 2.5 hours of education with Indonesia, Malaysia and Vietnam providing no sleep education at all ( Mindell *et al*., 2013). This lack of education could easily result in a generation of physicians who practice poor sleep hygiene, do not maintain consistent sleep wake schedules, and suffer from the health consequences of poor sleep quality, irregular sleep wake patterns and circadian rhythm misalignment. In addition, they do not optimize their learning capacity by getting appropriate sleep. Ultimately, these physicians may not be able to recognize, and thus manage, patients with treatable sleep disorders ( Haponik *et al.,* 1996, Rosen *et al.,* 1993). The advantages of including a sleep curricula of 2-4 hours of sleep education per year of medical school not only educates future doctors but contributes to increased well-being and decreased burn out among medical trainees themselves ( Salas *et al*., 2018).

To gain further insight on the inherent sleep patterns of medical students we sought to characterize the circadian rhythm sleep profile (chronotype) of medical students in a cross-cultural, ethnically diverse and international sample located in three different countries at different latitudes: Malaysia, the United States of America (USA) and the United Arab Emirates (UAE). Given that current studies have demonstrated that students living further away from the equator were more prone to an evening chronotype ( Miguel *et al*., 2014) and that the farther the distance the equator line equates with a more significant change towards an evening chronotype ( Leocadio-Miguel *et al.,* 2017) we anticipated that students in our study who lived closer to the equator (Malaysia) would demonstrate a morning chronotype when compared to those living further away (UAE and Baltimore).

## Methods

### Participants

**Figure 1.**
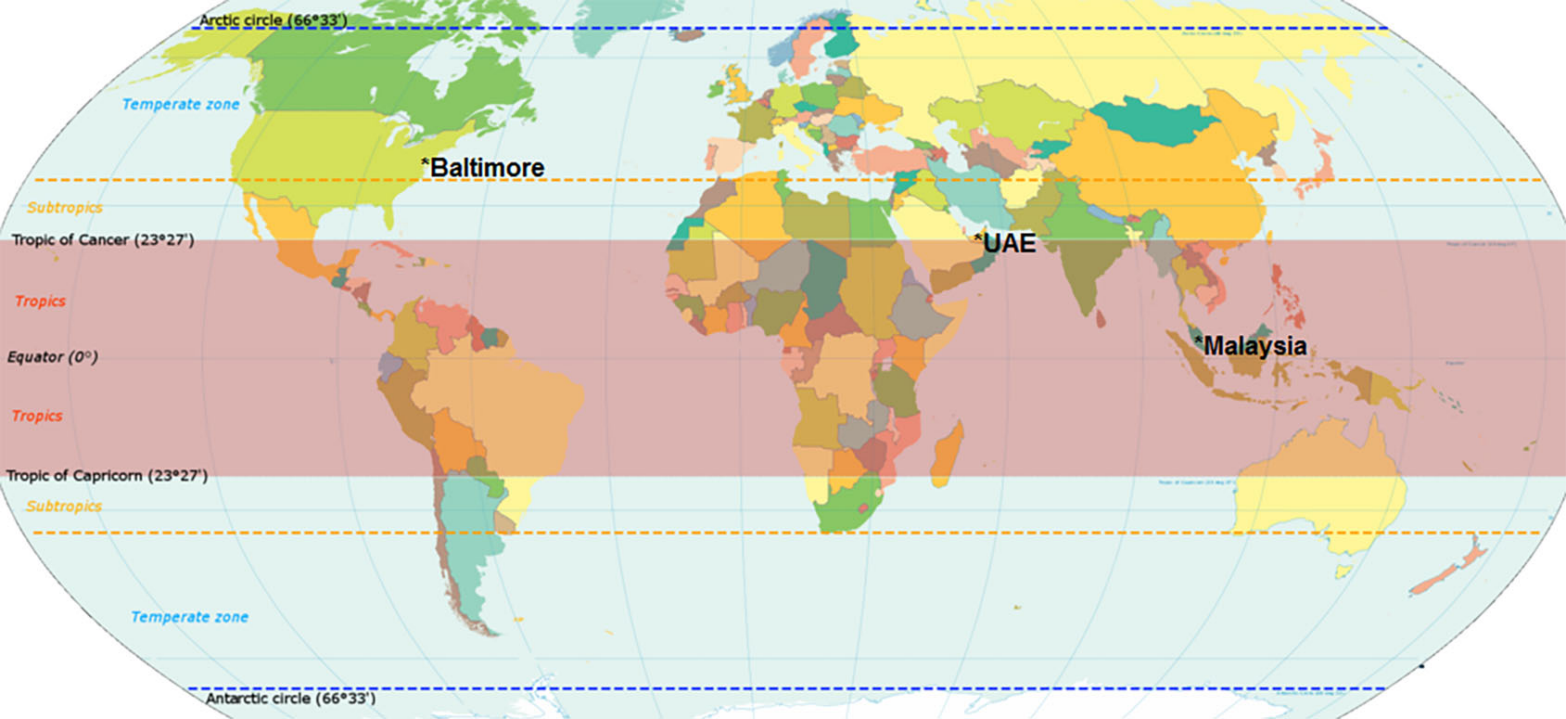
World map indicating study locations and their climate zones

Adapted from “ World map indicating tropics and subtropics” by KVDP (2013), licensed under CC BY-SA 3.0 / Study locations added to the original.

This study was a cross-sectional, questionnaire-based observational study conducted on three cohorts of medical students between May 2013 to May 2015 at Johns Hopkins School of Medicine (JHSOM), Perdana University (PU) in Malaysia and United Arab Emirates University (UAEU). Perdana University is a medical school in Kuala Lumpur with students of Indian, Chinese and Malay heritage. Kuala Lumpur is at latitude 3.13 °N and longitude 101.68 °E UAE (tropical -see Figure 1). UAEU is located in the city of Al Ain which is at latitude 24.207 °N and longitude 55.744 °E (Subtropical) and is a six-year medical school with 545 students (predominantly Emirati nationals). JHSOM is located in Baltimore, Maryland USA and is at latitude 39.28 °N and longitude 76.6 °W (Temperate).

The study population was comprised of 240 first year JHSOM students, 154 PU students and 545 UAEU students for a total of 939 medical students. At JHSOM the surveys were distributed during the students’ first year during the spring of 2014 and 2015. At PU the first three years of students were all given the questionnaires during the fall of 2013 and 2014. At UAEU the survey was given through an email to all six years of students during the spring of 2014. The study was approved by the local ethics committee in all three countries and written informed consent was obtained prior to any study procedures taking place. A total of 328 (35%) responses were obtained out of this study population. Participants ranged from 21 to 37 years of age.

### Questionnaire

The questionnaire was administered to a convenience sample of students and participation was voluntary. Information collected included class year, sex, ethnicity (PU only), and the Horne-Ostberg Morningness-Eveningness questionnaire. The Horne-Ostberg Morningness-Eveningness questionnaire is a validated questionnaire frequently used to assess the sleep-wake chronotype of individuals ( Horne and Ostberg, 1976). Participants are classified as *morning types* (wake up early, most alert early in the day) and *evening types* (most alert in the late evening hours with late bedtimes) or somewhere in-between. Based on the sum of all points in the questionnaire participants are classified as (1) Definitely morning type (70-86); (2) Moderately morning type (59-69); (3) Neither type (42-58); (4) Moderately evening type (31-41); and (5) Definitely evening type (16-30).

### Data Analysis

In preparation for data analysis, we assigned a number to each questionnaire we received. We then entered all the data in an Excel spreadsheet with unique codes for the cohort data (1 = PU, 2 = JHSOM, 3 = UAEU); gender data (male = 1, female = 2); and ethnicity data for the Malaysian students (1 = Indian, 2 = Chinese, 3 = Malay). We analyzed the data using SPSS Statistics ( IBM, 2017). Mann-Whitney U test and Kruskal-Wallis H test were used to compare groups by gender and ethnicity respectively. We used Kruskal-Wallis H test to analyze the chronotype score between the three cohorts as well as analyze the data for the individual items on the questionnaire ( Corder and Foreman, 2014). Post-hoc analysis for the individual questions was conducted using the Bonferroni adjustment.

## Results/Analysis

Of the 328 total questionnaires received, 10 were incomplete thus were not included in the analysis leaving 318 participants. Table 1 shows the number, sex, and age ranges of respondents from each cohort as well as the ethnicity for the PU and UAEU cohorts. Ethnicity data for the JHSOM cohort was not collected for individual students, however, ethnicity data for the two classes obtained from the registrar’s office is included in Table 1.

**Table 1.** Demographics of participants

	Class Year (n)	Sex (n)	Ethnicity (n)	Age Range (years)
JHSOM	First: 56	M: 25 F: 31	*Class demographics:* Asian: 83 American Indian/Alaskan Native: 4 Black/African American: 19 Hispanic: 20 Multiracial: 4 White: 101 Undetermined: 9	21-37
PU	First: 28 Second: 15 Third: 55	M: 11 F: 17 M: 6 F: 9 M: 22 F: 33	Indian: 7, Chinese: 10, Malay: 11 Indian: 3, Chinese: 10, Malay: 2 Indian: 14, Chinese: 29, Malay: 12	21-31
UAEU	First: 55 Second: 33 Third: 25 Fourth: 27 Fifth: 17 Six: 17	M: 28 F: 146	Emirati	17-27

### Difference between chronotypes of students in each university

**Figure 2.**
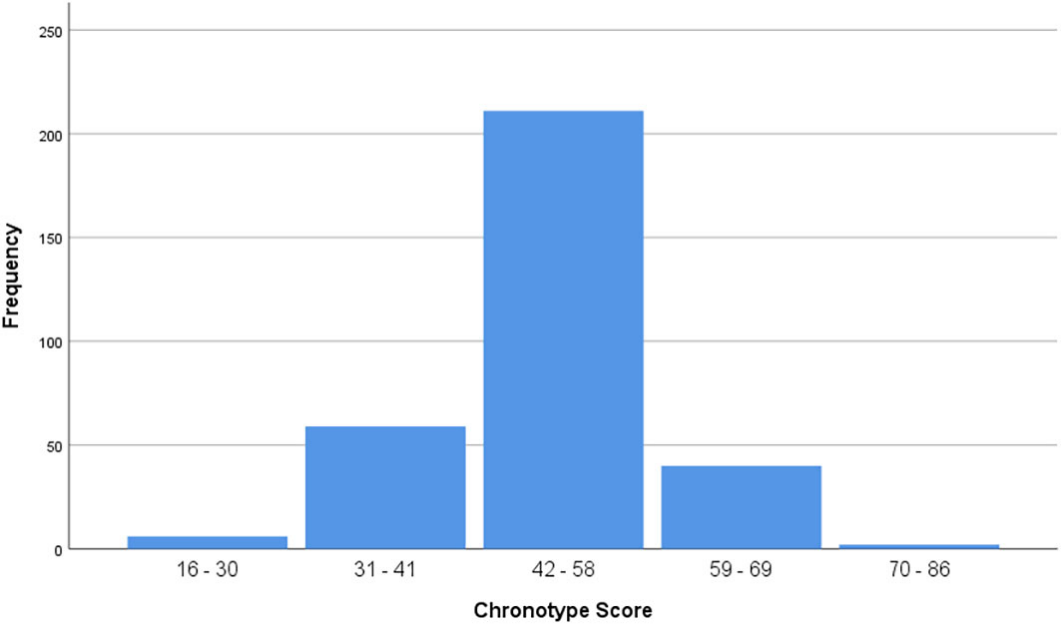
Frequency distribution of all chronotype scores

Overall, all three groups fell into the “neither” morning nor evening chronotype. Seeking to investigate if there was any statistical difference between the groups, we conducted a Kruskal-Wallis H test co-varied by group and found no significant difference in the chronotype score between the three groups ( *p* = 0.098). Table 2 presents the number of students in each chronotype category by cohort. Figure 2 shows a frequency distribution of the chronotype scores for all the three cohorts.

**Table 2.** Number of students in each chronotype category by cohort

	Definitely evening type(16-30)	Moderately evening type(31-41)	Neither Type(42-58)	Moderately morning type(59-69)	Definitely morning type(70-86)
JHSOM	0	12	38	5	1
PU	2	12	66	17	0
UAEU	4	35	107	18	1

### Difference in chronotype between gender

We sought to investigate if there was a significant difference between the genders in all three cohorts. We ran a Mann-Whitney U test of two independent samples and found no significant difference in the chronotype scores between male and female students. However, female students had higher chronotype scores compared to male students.

### Difference in chronotype between ethnicity in the Malaysian group

We ran a Kruskal-Wallis H test and found no significant difference in the chronotype score between the three ethnic groups in the Malaysian cohort ( *p* = 0.721).

### Difference in chronotype between years of study in the UAEU group

As noted earlier, the UAEU cohort differed slightly from the US and Malaysian in that the UAEU is a six-year program that students enter directly from high school as opposed to the US and Malaysian schools which are post-graduate studies following four years of undergraduate studies. A Kruskal-Wallis H test found no significant difference in chronotype score between the six years of students in the UAEU cohort ( *p* = 0.146).

### Statistically significant questions by location

We sought to investigate if there were any differences between the three cohorts in their responses to the 19 individual items on the questionnaire. We conducted a Kruskal-Wallis H test and found seven questions had a significant difference. Post-hoc analysis using the Bonferroni adjustment demonstrated the following findings:

Approximately what time would you get up if you were entirely free to plan your day?

There was a statistically significant difference between the waking times of the three cohorts of students ( *p* = 0.001). The Malaysian students got up earlier than the US and UAE students. Post-hoc analysis revealed the significant difference was between Malaysian and UAE ( *p* = 0.001) students. Malaysian and American students would choose to get up earlier in the morning if they were entirely free to plan their day as compared to the UAE students.

If you usually have to get up at a specific time in the morning, how much do you have to depend on an alarm clock?

All three cohorts of students were very much dependent on an alarm clock if they usually had to get up at a specific time in the morning. Although the median score was the same for the three groups, there was a significant difference between the three cohorts on how much they depended on an alarm clock ( *p* = 0.007). The difference was significant between Malaysian and UAE students ( *p* = 0.006).

How easy do you find it to get up in the morning (when you are not awakened unexpectedly)?

There was a significant difference between the three groups in how easy they found getting up in the morning when they were not woken up unexpectedly ( *p* = 0.040).The data showed that Malaysian students found it more easy to get up in the morning when they were not awakened unexpectedly compared to US and UAE students. The difference was significant, however, only between the Malaysian and UAE students ( *p* = 0.045).

If you had no commitments the next day, what time would you go to bed compared to your usual bedtime?

The findings revealed a significant difference between the three cohorts in the time the students would go to bed compared to their usual bedtime if they had no commitments the next day ( *p* = 0.023). Overall, all three groups were likely to go to bed more than 1- 2 hours later than their normal bedtimes. However, further analysis showed the difference was significant between US and UAE students ( *p* = 0.020).

You have two hours of hard physical work. You are entirely free to plan your day. Considering only your internal “clock”, which of the following times would you chose?

According to the results, there was a statistically significant difference between the three cohorts ( *p* = 0.007). Post-hoc analysis revealed the significant difference was between the UAE and Malaysian students ( *p* = 0.016). UAE students were more likely to do hard physical work later in the day compared to the US and Malaysian students.

At approximately what time of day do you usually feel your best?

There was a significant difference between the cohorts in the time of day they usually felt their best ( *p* = 0.011). Further analysis showed that the difference was significant between Malaysian and US students ( *p* = 0.039) as well as UAE students ( *p* = 0.021). All three groups had a median score of 3.

One hears about “morning types” and “evening types.” Which one of these types do you consider yourself to be?

The findings revealed a statistically significant difference between the three student groups ( *p* = 0.001). This difference was significant for Malaysian students compared to US students ( *p* = 0.001) and UAE students ( *p* = 0.017). Malaysian students were more likely to consider themselves a “morning type” while US students and UAE students were more likely to consider themselves an “evening type”.

Statistically significant questions by gender

Similarly, we conducted a Mann-Whitney U test on the individual questions with males and females as the two independent samples and found five questions had a significance difference. The five questions are presented below:

Approximately what time would you go to bed if you were entirely free to plan your evening?

There was a statistically significant difference between the time male and female students would go to bed if they were free to plan their evening ( *p* = 0.007). Female students chose to go to bed earlier than male students.

If you usually have to get up at a specific time in the morning, how much do you have to depend on an alarm clock?

Both female and male students were very dependent on an alarm clock if they had to get up at a specific time in the morning.There was a significant difference, however, in how much the male and female students depended on an alarm clock ( *p* = 0.017). Male students were, however, less dependent on an alarm clock than female students.

At approximately what time in the evening do you feel tired, and, as a result, in need of sleep?

The findings revealed a significant difference between male and female students in the time they felt tired in the evening and needed to sleep ( *p* = 0.021). Based on the mean ranks, female students felt tired and went to bed earlier in the evening than male students.

You want to be at your peak performance for a test that you know is going to be mentally exhausting and will last for two hours. You are entirely free to plan your day. Considering only your own internal “clock,” which one of the four testing times would you choose?

There was a statistically significant difference between the testing times female and male students chose considering only their internal clock ( *p* = 0.005). Female students chose testing times that were earlier in the day while male chose later testing times.

You have decided to engage in hard physical exercise. A friend suggests that you do this for one hour twice a week. The best time for him/her is between 10-11 PM (22-23 h). Bearing in mind nothing else but your own internal “clock,” how well do you think you would perform?

Both male and female students would be in reasonable form if they were to engage in hard physical exercise for one hour twice a week between 10-11 PM. However, the results revealed a significant difference in how well the male and female students would perform ( *p* = 0.007). Female students found it difficult to perform well than male students.

## Discussion

Given that prior studies on chronotype and latitude have demonstrated that students living further away from the equator were more prone to an evening chronotype ( Miguel *et al.,* 2014) and that the farther the distance the equator line equates with a more significant change towards an evening chronotype ( Leocadio-Miguel *et al*., 2017) we sought to determine whether location, ethnicity and gender influenced the chronotype of medical students living closer to the equator (Malaysia) compared to those living at higher latitudes (UAE and Baltimore).

The results of our study showed no significant difference in the chronotypes of the UAE, Malaysian and United States medical students when looking at composite scores on the Horne-Ostberg survey. However, scores did show that UAE students trended slightly more towards evening types and an analysis looking at individual question responses in the survey did reveal significant differences in student responses across the three medical schools for habitual bedtime, wake time and preferred hours for performing work that suggests circadian preference patterns. On average, students in the UAE ideally preferred to wake up later in the day and do “hard physical work” at later times in the day. This corresponds with their higher dependence on an alarm clock to get up in the morning. One might surmise that this fits with a desert Bedouin society that is traditionally nocturnal given the extremely high daytime temperatures in this region.

We also found no statistically significant difference in chronotype scores by gender. This is comparable to another larger study that found no significant difference by gender ( Paine *et al.,* 2006). The mean ranks revealed, however, that male students had lower chronotype scores, thus, trending more towards an evening chronotype. This notion is supported by the results of individual questions that had a significant difference by gender. Male students chose to go to bed later, chose later testing times, and performed well during hard physical exercise later in the evening. Four of the five questions that had a significant difference by gender are similar to results reported in a different larger study ( Adan and Natale, 2002). The authors of this study found 12 questions had a significant difference by gender. We might have found more questions with a significant difference by gender had our sample size been larger.

### Latitudinal differences

While all three cohorts overall fell into the “neither” morning nor evening chronotype, sub question analysis demonstrated a clear and significant propensity for Malaysian students to rise earlier than the US or UAE students and to “feel their best earlier in the day” compared to US and UAE students. They were also more likely to consider themselves a “morning type” compared to US and UAE students. These results lend support to prior studies showing a shift towards “morningness” chronotypes in those individuals living closer to the equator ( Miguel *et al.*, 2014). In Brazil, students ( Miguel *et al*., 2014) and subjects ( Leocadio-Miguel *et al.,* 2017) living further away from the equator were more prone to an evening chronotype and university students in six countries demonstrated the same results ( Smith *et al.,* 2002). Our results are a partial replication of these prior studies demonstrating differences in chronotype according to latitude however focusing on medical students - a population specifically vulnerable to sleep abnormalities. It is possible that increasing the sample size of our cohorts would increase the significant findings within the MEQ to give a more robust significant result.

### Interventional strategies - sleep education

Educating young physicians about sleep, sleep hygiene and sleep deprivation may be one of the most effective interventions to combat poor sleep practices among this highly susceptible group ( Salas *et al.,* 2013, Gamaldo and Salas, 2008). The sleep habits of medical students are important considering the health and psychiatric consequences of sleep deprivation for both the students and the safety of patients they will ultimately care for. Recent evidence also suggests higher risk of depression among individuals with circadian phase delay chronotype which may serve as an important area to target student health and wellbeing screening strategies ( Murray *et al*., 2017). Medical trainees across the globe will inevitably encounter patients with sleep difficulties. Failure to address the detrimental personal sleep practices often unconsciously practiced by medical students could potentially result in lack of recognition of these same or related sleep disorders in their patients. A recent consensus among leading sleep medicine and neurology educators has proposed a proactive evidence-based initiative to span sleep medicine education throughout each year of the medical school curriculum to effectively ingrain the importance of sleep hygiene and sleep medicine within the next generation of medical professionals ( Salas *et al*., 2018).

### Limitations

Our study extends the knowledge of sleep chronotype characterization in a diverse group of medical students as well as provides a cross-cultural and geographical comparison of chronotype presentation between students. There are some limitations to our study including possible selection and self-reporting bias, challenges with generalizability to other medical students and schools and the inability to accurately record changes in sleep behavior over time which results from the cross-sectional study design. Having only one measure of chronotype - self reporting is less than ideal and having biological measures such as actigraphy, temperature or melatonin measurements would have been preferable.

It remains challenging to determine whether our three cohorts are comparable for several reasons. First, the US medical school group is comprised solely of first year medical students whereas approximately two thirds of the Malaysian cohort were in their first and second (pre-clinical) years. The students in the UAE were from all 1-6 years of medical school and did not show a significant difference in chronotype. Second, demands on students can clearly differ between institutions. For example, the Malaysian students began their mandatory classes at 9 am whereas the US students began at 8 am (although these 8 am lectures are not mandatory and are watched at home during their own time by more than half the class). These different schedules could affect chronotype by forcing students to waken earlier than they ordinarily would.

We propose that increasing student exposure to sleep medicine ultimately improves not only physician self-awareness of sleep health and hygiene but that of their patients, thereby decreasing future medical errors and misdiagnoses and increasing health outcomes both domestically and abroad.

## Conclusion

Overall, neither medical school location (based on latitude) nor student demographics (nationality and ethnicity) appeared to significantly affect reported sleep chronotype in medical students from the United States, UAE and Malaysia however, some individual questions on the chronotype questionnaire demonstrated a significant difference in that Malaysian students rose earlier, felt better earlier in the day and considered themselves more “morning-types” compared to the US and UAE students. Sleep chronotype and habits among medical students and physicians can ultimately result in greater awareness of sleep abnormalities in patients ultimately improving patient safety, care and wellbeing and decreasing burn out among medical professionals themselves ( Salas *et al.,* 2018).

## Take Home Messages

• A cross-sectional study was conducted on 328 medical students in three countries to determine if demographics or latitude affect chronotype.
• Sub question analysis demonstrated Malaysian students rise earlier and consider themselves a “morning type” compared to US and UAE.
• Sleep education is an effective intervention to combat poor sleep practices, ultimately improving physician well-being, patient care and safety.

## Notes On Contributors

Nicoline Schiess MD, MPH: Dr. Schiess is an Assistant Professor of Neurology at Johns Hopkins University, Director of the Neuroscience course for Hopkins medical students and Co-Director for the Johns Hopkins Global Neurology program. An international educator, she has taught globally in programs in the UAE, Malaysia, Zambia and Haiti. ORCID: https://orcid.org/0000-0002-0121-8453

Violet Kulo, MS, EdD: Dr. Violet Kulo is an instructional designer at the Johns Hopkins School of Medicine. She received her doctorate in education from Lehigh University in Pennsylvania, USA. She manages program evaluation and assessments in the pre-clinical curriculum. ORCID: https://orcid.org/0000-0002-5386-9549

Jennifer L. Dearborn MD, MPH: Dr. Dearborn completed her residency in Neurology and fellowship in Vascular Neurology at the Johns Hopkins School of Medicine. She is an Instructor of Neurology at Harvard Medical School and is affiliated with Beth Israel Deaconess Medical Center. ORCID: https://orcid.org/0000-0001-6733-1072

Sami Shaban PhD: Dr. Sami Shaban is an Associate Professor in the College of Medicine and Health Sciences, UAE University. He received a PhD in Health Informatics from the Medical University of South Carolina, USA. He teaches Health Informatics courses including Healthcare Database Administration, Health Information Applications and Healthcare Informatics. ORCID: https://orcid.org/0000-0003-0312-4722

Charlene E. Gamaldo MD, FAAN, FAASM: Dr. Charlene Gamaldo is the medical director of the Johns Hopkins Center for Sleep at Howard County General Hospital. Her research interests are in the area of sleep medicine, specifically investigating the co-morbid health consequences of chronic sleep deprivation. ORCID: https://orcid.org/0000-0002-9386-9666

Rachel Marie E. Salas MD, MedHP, FAAN: Dr. Rachel Salas is an Associate Professor of Neurology at Johns Hopkins. She is the Co-Director of the Neurology Core Clerkship for Medical students and is the Co-Director for the Neurology resident and Neurophysiology Fellow JHH Sleep Rotation. ORCID: https://orcid.org/0000-0002-3945-3336

## References

[ref1] Adan A. and Natale V. (2002) Gender differences in morningness-eveningness preference. Chronobiology International. 19(4), pp.709–720. https://doi.org/10.1081/CBI-120005390 12182498 10.1081/cbi-120005390

[ref2] Ahmed N. Sadat M. and Cukor D. (2017) Sleep Knowledge and Behaviors in Medical Students: Results of a Single Center Survey. Academic Psychiatry. 41(5), pp.674–678. https://doi.org/10.1007/s40596-016-0655-3 28097529 10.1007/s40596-016-0655-3 PMC5513792

[ref3] Ayala E.E. Berry R. Winseman J.S. and Mason H.R. (2017) A cross-sectional snapshot of sleep quality and quantity among US medical students. Academic Psychiatry. 41(5), pp.664–668. 10.1007/s40596-016-0653-5 28091977

[ref4] Ayalon R.D. and Friedman F. (2008) The effect of sleep deprivation on fine motor coordination in obstetrics and gynecology residents. American Journal of Obstetrics and Gynecology. 199(5), pp.576.e1–576.e5. 10.1016/j.ajog.2008.06.080 18822404

[ref5] Beebe D.W. Rose D. and Amin R. (2010) Attention, learning, and arousal of experimentally sleep-restricted adolescents in a simulated classroom. The Journal of Adolescent Health. 47(5), pp.523–525. 10.1016/j.jadohealth.2010.03.005 20970088 PMC2963797

[ref6] Bleich S.N. Bandara S. Bennett W.L. Cooper L.A. (2014) Impact of non-physician health professionals. BMI on obesity care and beliefs’, Obesity. 22(12), pp.2476–2480. 10.1002/oby.20881 25185506 PMC4236247

[ref7] Corder G.W. and Foreman D.I. (2014) Nonparametric Statistics: A Step-by-Step Approach. 2nd edn. Hoboken, New Jersey: Wiley.

[ref8] Curcio G. Ferrara M. and De Gennaro L. (2006) Sleep loss, learning capacity and academic performance. Sleep Medicine Reviews. 10(5), pp.323–337. 10.1016/j.smrv.2005.11.001 16564189

[ref9] Diekelmann S. and Born J. (2010) The memory function of sleep. Nature Reviews Neuroscience. 11(2), pp.114–126. 10.1038/nrn2762 20046194

[ref10] Eliasson A.H. Lettieri C.J. and Eliasson A.H. (2010) Early to bed, early to rise! Sleep habits and academic performance in college students. Sleep Breath. 14(1), pp.71–75. 10.1007/s11325-009-0282-2 19603214

[ref11] Ferrara M. Iaria G. De Gennaro L. Guariglia C. (2006) The role of sleep in the consolidation of route learning in humans: a behavioural study. Brain Research Bulletin. 71(1-3), pp.4–9. 10.1016/j.brainresbull.2006.07.015 17113921

[ref12] Gamaldo C.E. and Salas R.E. (2008) Sleep medicine education: are medical schools and residency programs napping on the job? Nature Clinical Practice Neurology. 4(6), pp.344–345. 10.1038/ncpneuro0808 18446150

[ref13] Genzel L. Ahrberg K. Roselli C. Niedermaier S. (2013) Sleep timing is more important than sleep length or quality for medical school performance. Chronobiology International. 30(6), pp.766–771. 10.3109/07420528.2012.763132 23750895

[ref14] Grady F. and Roberts L.W. (2017) Sleep deprived and overwhelmed: Sleep behaviors of medical students in the USA. Academic Psychiatry. 41(5), pp.661–663. 10.1007/s40596-017-0804-3 28913770

[ref15] Haponik E.F. Frye A.W. Richards B. Wymer A. (1996) Sleep history is neglected diagnostic information. Challenges for primary care physicians. Journal of General Internal Medicine. 11(12), pp.759–761. 10.1007/BF02598994 9016425

[ref16] Horne J.A. and Ostberg O. (1976) A self-assessment questionnaire to determine morningness-eveningness in human circadian rhythms. International Journal of Chronobiology. 4(2), pp.97–110. 1027738

[ref17] IBM (2017) SPSS Statistics. Version 25. Armonk, NY.

[ref18] Krause A.J. Simon E.B. Mander B.A. Greer S.M. (2017) The sleep-deprived human brain. Nature Reviews Neuroscience. 18(7), pp.404–418. 10.1038/nrn.2017.55 28515433 PMC6143346

[ref19] KVDP (2013) World map indicating tropics and subtropics. Available at: https://commons.wikimedia.org/wiki/File:World_map_indicating_tropics_and_subtropics.png( Accessed: 29 May 2018).

[ref20] Leocadio-Miguel M.A. Louzada F.M. Duarte L.L. Areas R.P. (2017) Latitudinal cline of chronotype. Scientific Reports. 7(1), pp.5437. 10.1038/s41598-017-05797-w 28710358 PMC5511182

[ref21] Miguel M. Oliveira V.C. Pereira D. and Pedrazzoli M. (2014) Detecting chronotype differences associated to latitude: a comparison between Horne--Ostberg and Munich Chronotype questionnaires. Annals of Human Biology. 41(2), pp.105–108. 10.3109/03014460.2013.832795 24059265

[ref22] Mindell J.A. Sadeh A. Kwon R. and Goh D.Y. (2013) Cross-cultural differences in the sleep of preschool children. Sleep Medicine. 14(12), pp.1283–1289. 10.1016/j.sleep.2013.09.002 24269649

[ref23] Murray J.M. Sletten T.L. Magee M. Gordon C. (2017) Prevalence of circadian misalignment and its association with depressive symptoms in delayed sleep phase disorder. Sleep. 40(1). 10.1093/sleep/zsw002 28364473

[ref24] Owens J. (2005) Introduction to special section: NIH Sleep Academic Award program. Sleep Medicine. 6(1), pp.45–46. 10.1016/j.sleep.2004.11.001 15680295

[ref25] Paine S.J. Gander P.H. and Travier N. (2006) The epidemiology of morningness/eveningness: influence of age, gender, ethnicity, and socioeconomic factors in adults (30-49 years). Journal of Biological Rhythms. 21(1), pp.68–76. 10.1177/0748730405283154 16461986

[ref26] Peigneux P. Laureys S. Delbeuck X. and Maquet P. (2001) Sleeping brain, learning brain. The role of sleep for memory systems. Neuroreport. 12(18), pp.A111–24. 10.1097/00001756-200112210-00001 11742260

[ref27] Pereira D.S. Tufik S. Louzada F.M. (2005) Association of the length polymorphism in the human Per3 gene with the delayed sleep-phase syndrome: does latitude have an influence upon it? Sleep. 28(1), pp.29–32. 10.3410/f.1024974.294734 15700718

[ref28] Preckel F. Lipnevich A.A. Boehme K. Brandner L. (2013) Morningness-eveningness and educational outcomes: the lark has an advantage over the owl at high school. The British Journal of Educational Psychology. 83(Pt 1), pp.114–134. 10.1111/j.2044-8279.2011.02059.x 23369178

[ref29] Rosen R.C. Rosekind M. Rosevear C. Cole W.E. (1993) Physician education in sleep and sleep disorders: a national survey of U.S. medical schools. Sleep. 16(3), pp.249–254. 10.1093/sleep/16.3.249 8506458

[ref30] Salas R.E. Gamaldo A. Collop N.A. Gulyani S. (2013) A step out of the dark: improving the sleep medicine knowledge of trainees. Sleep Medicine. 14(1), pp.105–108. 10.1016/j.sleep.2012.09.013 23127578

[ref31] Salas R.M.E. Strowd R.E. Ali I. Soni M. (2018) Incorporating sleep medicine content into medical school through neuroscience core curricula. Neurology. 91(13), pp.597–610. 10.1212/WNL.0000000000006239 30185444

[ref32] Smith C.S. Folkard S. Schmieder R. Parra L.F. (2002) Investigation of morning-evening orientation in six countries using the preferences scale. Personality and Individual Differences. 32, pp.949–968. 10.1016/S0191-8869(01)00098-8

[ref33] Smith C. (1996) Sleep states, memory processes and synaptic plasticity. Behavioural Brain Research. 78(1), pp.49–56. 10.1016/0166-4328(95)00218-9 8793037

[ref34] Wolfson A.R. and Carskadon M.A. (2003) Understanding adolescents’ sleep patterns and school performance: a critical appraisal. Sleep Medicine Reviews. 7(6), pp.491–506. 10.1016/s1087-0792(03)90003-7 15018092

