# Supplementary material for: Cross-Cultural Chronotypes: Educating Medical Students in America, Malaysia and the UAE
Source: MedEdPublish (2016). 2020 Jan 7;9:5. [Version 1] doi: 10.15694/mep.2020.000005.1 (PMC10697528; doi:10.15694/mep.2020.000005.1)
Supplement: Peer review history

# Reviewer report: Reviewer response for version 1

**DOI:** 10.21956/mep.19958.r30322

**Published:** 2020-04-13

**Authors:** P Ravi Shankar

**Affiliations:** 1 American International Medical University

This review has been migrated. The reviewer awarded 4 stars out of 5 This is an interesting and well-written manuscript. The concept of ‘chronotype’ was a new one for me. I also learned about the variation in chronotype with latitude. In countries father away from the equator there will be more seasonal variations in day light. Does the longer nights during winter affect the chronotype? I am aware about the practice of siesta which is common in many subtropical countries and countries around the Mediterranean. The practice of sleeping in the afternoon leads to delay in going to bed at night. Individuals may want to take advantage of the cooler nights and avoid the summer heat. In the UAE I believe sleeping habits may be different during the fasting holy month of Ramadan. The methodology, results and limitations of the study are well described. Is there any educational implications of different chronotypes? Should classes start later in a student population with a predominantly evening chronotype? I agree with the authors that sleep hygiene and proper sleep habits are not emphasized in the medical curriculum. Proper sleep may be important for physical and mental well-being. As societies develop and artificial lighting becomes more common, both the time of waking up and going to bed get delayed. I believe human beings have shifted more toward an evening phenotype over the last four centuries? It will be interesting to know the participation rates at the three institutions. The authors can consider providing more information about the Horne-Ostberg Morningness-Eveningness questionnaire . The article will be of board interest especially the educational implications of strengthening sleep education.

Reviewer Expertise:

NA

No decision status is available

# Reviewer report: Reviewer response for version 1

**DOI:** 10.21956/mep.19958.r30323

**Published:** 2020-03-28

**Authors:** Ken Masters

**Affiliations:** 1 Sultan Qaboos University

This review has been migrated. The reviewer awarded 3 stars out of 5 The paper deals with cross-cultural chronotypes of students in USA, Malaysia and the UAE. Overall, the paper is well written. It begins by explaining the problems of lack of sleep in medical students and doctors, and also a central idea – that people located further away from the equator tend to be an evening chronotype more than people located closer to the equator. The experiment and testing have been well conducted, and the results well presented.An area of weakness in the paper, however, is the relationship between the study and medical education. After the Results have been given in detail, the Discussion tends to repeat and summarise the results (with a bit of reflecting to the current literature), and then notes that there were differences between the groups (perhaps not as clear-cut as one would expect). And then there is a sudden jump to a discussion about the importance of educating medical students about the importance of sleep hygiene and sleep deprivation. But what appears to be missing is a discussion about the significance of these findings for medical education. How does this study impact on our knowledge, and how should we take it into account in our medical education?The link may be obvious to the authors, but, given that it is an under-studied area (an important point made in the paper), the link will not be obvious to most readers. (The Abstract’s conclusion hints at it, but this is not described enough in the Discussion). By not making that link, the paper may be perceived by readers to be a mildly interesting survey of medical student sleep preferences, but the significance of which is not clear. Essentially, the authors need to directly address the “So what?” of their study.I look forward to a Version 2 of the paper in which this is clarified.

Reviewer Expertise:

NA

No decision status is available

# Reviewer report: Reviewer response for version 1

**DOI:** 10.21956/mep.19958.r30321

**Published:** 2020-02-07

**Authors:** Barbara Jennings

**Affiliations:** 1 Norwich Medical School

This review has been migrated. The reviewer awarded 4 stars out of 5 A consideration of circadian regulation and chronotypes is not a mainstream topic for medical educators, and I have no expertise in the underlying physiology behind this phenomenon. However, this well written article caught my attention and interest.There are many good points to make about this paper including the citation list, the method descriptions and the standard of research governance for the project. The authors clearly describe what they did and how the data was analysed, including the sensible use of statistical adjustment in the face of multiple comparisons. They also declare limitations to their study design in their Discussion: in particular, they consider how comparable their cohorts are. Questions I have for the authors include – did you have a primary aim for the quantitative aspects of the study, and could you have carried out power calculations to determine confidence levels for the conclusions drawn (and negative findings in particular?)This article may be of interest to educators who lead aspects of professionalism or professional well being, and even those whose concerns include rota and timetable management.

Reviewer Expertise:

NA

No decision status is available
